# Functional characterization of a gibberellin receptor and its application in alfalfa biomass improvement

**DOI:** 10.1038/srep41296

**Published:** 2017-01-27

**Authors:** Xuemin Wang, Jun Li, Liping Ban, Yudi Wu, Xinming Wu, Yunqi Wang, Hongyu Wen, Vladimir Chapurin, Nikolay Dzyubenko, Zhiyong Li, Zan Wang, Hongwen Gao

**Affiliations:** 1 Institute of Animal Sciences, Chinese Academy of Agricultural Sciences, Beijing 100193, China; 2 Institute of Grassland Research, Chinese Academy of Agricultural Sciences, Hohhot 010010, China; 3 College of Animal Science and Technology, China Agricultural University, Beijing 100193, China; 4 Animal Husbandry and Veterinary institute, Shanxi Academy of Agricultural Sciences, Taiyuan 030032, China; 5 N.I.Vavilov All-Russian Research Institute of Plant Industry, St. Petersburg 190000, Russia

## Abstract

Bioactive gibberellins (GAs) are essential phytohormones involved in the regulation of many aspects of plant development. GA receptors are crucial in GA signal transduction in plants. The GA receptor *GoGID1* promotes plant elongation and improves biomass production when ectopically expressed in tobacco. Here, we discovered that *GoGID1* can interact with the DELLA proteins of *Arabidopsis* in the presence of gibberellic acid. *GoGID1* partially or completely functionally rescued the phenotypes of the *Arabidopsis* double-mutants *atgid1a*/*atgid1c* and *atgid1a*/*atgid1b*. The overexpression of *GoGID1* led to increases in plant height and biomass production in transgenic *Arabidopsis* plants. The *GoGID1* gene enhanced GA sensitivity of the transgenic plants. More importantly, transgenic alfalfa plants overexpressing *GoGID1* exhibited increased growth rates, heights and biomass and produced larger leaves when compared with the control plants. Thus, *GoGID1* functions as a GA receptor, playing multiple roles in plant growth and development. The *GoGID1* gene has the potential to be used in the genetic engineering of forage crops for biomass improvement.

Gibberellins (GAs) are well-known plant hormones that participate in the regulation of many growth and developmental processes in plants^1^,^2^, including seed germination, stem elongation, leaf expansion, pollen maturation and flower induction^2^. Research over the past few years has elucidated the molecular mechanisms of GA perception and signaling in plants. Generally, the GA signal is recognized by the GA receptor, GA INSENSITIVE DWARF1 (GID1), which is a soluble protein localized to both the cytoplasm and nucleus. The GA-GID1 complex has the ability to interact with DELLA proteins. DELLA proteins are nuclear transcriptional regulators that function as pivotal negative regulators in the GA-signaling cascade. GA-GID1-DELLA binding results in the rapid degradation of DELLAs through the proteasome pathway, which launches the GA reaction.

GA receptor protein(s), including GIDs, are the primary factors mediating GA perception in mono- and dicotyledonous plants^3^. The first GA receptor was identified in rice by studying GID mutants in 2006^4^. Subsequently, GA receptors were identified in many plants, such as *Arabidopsis*^5^, cotton^6^, barley^7^ and *Galega orientalis*^8^. *Arabidopsis* has three GID1 orthologs (GID1A, GID1B and GID1C), and each of the three AtGID1 proteins interacts with each of the five AtDELLA proteins [including *GA INSENSITIVE* (GAI), *REPRESSOR OF ga1*-*3* (RGA), *RGA*-*LIKE1 (RGL1*), RGL2 and RGL3]^9^. The interaction between DELLA and GID1 is presumably a precursor to GA signal transduction. Upon GID1-DELLA protein interaction, the DELLA protein is recognized by the F-box subunit of an SCF E3 ubiquitin ligase, such as *SLY1 (SLEEPY1*) in *Arabidopsis* or GID2 in rice^10^,^11^,^12^. The GID1-GA-DELLA complex stimulates a protein-protein interaction between DELLA and SLY1, which poly-ubiquitinates DELLA proteins, thereby targeting them for degradation by the 26 S proteasome pathway^10^,^12^.

GID loss-of-function mutations partly or completely shut down GA signaling, and the mutants show severe dwarfing phenotypes or low fertility levels^4^,^13^. The recessive rice *gid1* mutant shows a typical GA-insensitive phenotype^4^. In *Arabidopsis*, none of the loss-of-function mutants have apparent phenotypes, while the double-knockout mutants show various phenotypes. The double mutant *atgid1a*/*atgid1c* showed a dwarf phenotype. The stamens of the double-mutant *atgid1a*/*atgid1b* were significantly shorter than those of the wild-type plant, resulting in a lower seed yield, and an irregular surface pattern^13^, which indicated that the three orthologs have distinct functions in regulating different developmental processes.

Biomass yield is a highly complex trait and various approaches have been applied to improve this important trait. Plant hormones, especially brassinosteroids, auxins, GAs and cytokinins, regulate plant growth processes and play pivotal roles in biomass production^14^,^15^. In the 1960s, the so called rice ‘green revolution’ largely improved rice yields using the semi-dwarf variety of rice IR8. The IR8 phenotype was caused by the *sd1* gene, which encodes an oxidase enzyme involved in the plant’s GA biosynthesis^16^.

GAs are important determinants of plant height. Improvements in biomass yield have been achieved by altering GA metabolism^17^ and signaling^8^ in tobacco. The overproduction of *OsGID1* in transgenic rice revealed a GA-overdose phenotype, resulting in tall plants with long leaves^4^. The overexpression of *PttGIDs* resulted in taller stems and larger rosettes in transgenic *Arabidopsis*, and transgenic aspen grew taller^18^. The overexpression of *GoGID1* in transgenic tobacco plants also promoted plant elongation and improved biomass production^8^. Biomass production is the most important trait in forage crops. However, there have been limited studies on the relationship between GA and biomass in forage crops.

Alfalfa (*Medicago sativa* L.), an important forage crop, is critical to the livestock industry and to sustainable agriculture worldwide. However, alfalfa yield improvements have lagged behind those of many other crops^19^,^20^. The genetic enhancement of alfalfa, increasing its biomass, could have profound impacts on the forage industry. The GA receptor gene *GoGID1* was initially isolated from *Galega orientalis*^8^, a perennial legume that is closely related to alfalfa. The *GoGID1* gene can improve biomass production in transgenic tobacco^8^. In this study, we further demonstrated that *GoGID1* encodes a biologically functional GA receptor and can increase biomass in transgenic alfalfa. This transgenic approach is valuable for developing high biomass cultivars of forage crops.

## Results

### The tissue-specific expression analysis

**Figure 1:**
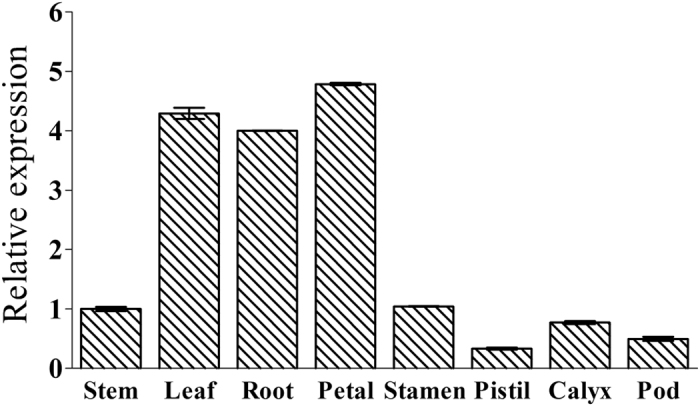
Expression profile of GoGID1 in *G. orientalis* tissues. Samples of different organs were collected from 2-year-old G. *orientalis* plants. The relative transcript levels of the gene in different tissue samples were determined by quantitative real-time PCR. *GoActin* was used as the internal reference. The bars show the mean of triplicates ± SD.

The quantitative real-time-PCR (qRT-PCR) method was used to elucidate the expression profile of *GoGID1* in different *G. orientalis* tissues, including stems, leaves, roots, petals, stamens, pistils, calyces and pods. As shown in Fig. 1, while *GoGID1* is constitutively expressed in all of the tissues tested, the transcript levels were highest in the petals, followed by leaves and roots, and the pistils had the lowest expression level.

### Interaction of GA-signaling components *in vivo*

**Figure 2:**
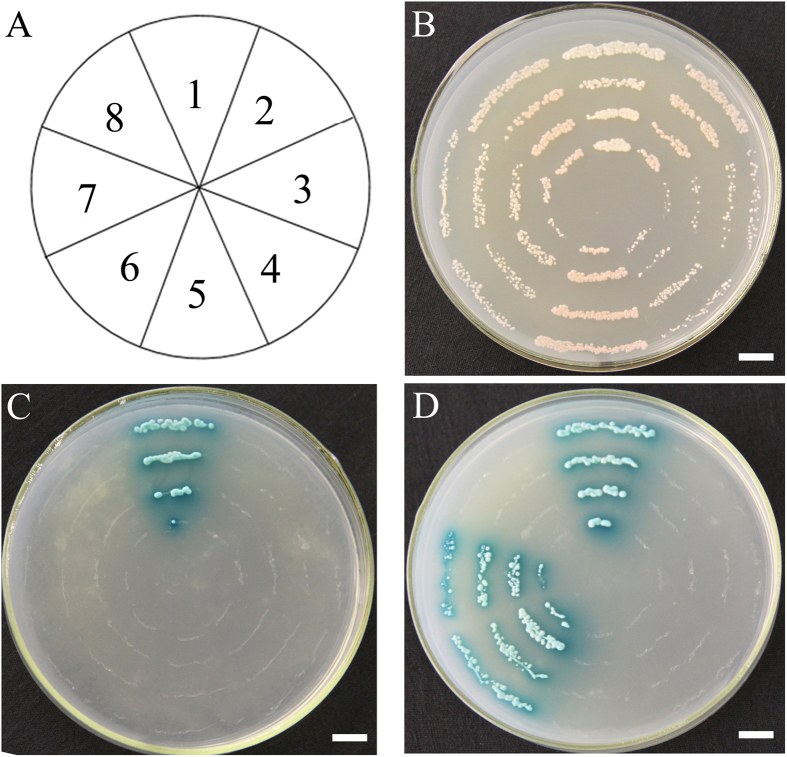
Interaction of GoGID1-AtDELLA in a Y2H assay. GoGID1 was used as bait, and either AtRGA or AtGAI was used as prey, bars:1 cm. (**A**) Nos 1 through 8 indicate plasmid combinations as follows:1, positive control; 2, AD/BD; 3, AD-AtRGA/BD; 4, AD-AtGAI/BD; 5, AD/BD-GoGID1; 6, AD-AtRGA/BD- GoGID1; 7, AD-AtGAI/BD-GoGID1; 8, negative control. (**B**) The same colonies on DDO medium (double-dropout medium: SD/–Leu/–Trp). (**C**) The same colonies on QDO/X/A medium (quadruple-dropout medium: SD/–Ade/–His/–Leu/–Trp supplemented with X-α-Gal and aureobasidin **A**) without GA_3_. (**D**) The same colonies on QDO/X/A medium with 10^−4^ M GA_3_.

The GA-dependent binding of the GA receptor to a DELLA protein is a fundamental requirement for GA signal transduction in mono- and dicotyledonous plants^4^,^5^. The GID-DELLA interaction results in the degradation of RGA or GAI in *Arabidopsis*. To verify that the *G. orientalis* GID1 protein has GA receptor activity and the ability to interact with DELLA proteins, we assessed the binding between GoGID1 and RGA or GAI from *Arabidopsis* (based on the high homology between GoGID1 and AtGIDs) using the yeast two-hybrid system. In this assay, the bait plasmid contained the *G. orientalis* GID1-coding sequence fused with the Gal4 DNA-binding domain (BD). The *Arabidopsis* RGA- and GAI-coding sequences were fused with the Gal4 transcriptional activation domain (AD) in the prey plasmid. Then, these two fusion proteins were co-transformed into the yeast strain AH109. Figure 2D shows that the transformation of bait or prey plasmids individually did not rescue cell growth in either the presence or absence of GA [10^−4^ M gibberellic acid (GA_3_)]. Yeast cells carrying the plasmid pBD-GoGID1 with either pAD-RGA or pAD-GAI grew well on SD/-Ade/-His/-Leu/-Trp/X-a-Gal (QDO + X-a-Gal) agar plates in the auxotrophic screen and showed LacZ activity in the presence of GA_3,_ similar to the positive controls (Fig. 2D). However, the yeast cells containing the plasmid combinations of pBD with DELLA [either pAD-RGA(3) or pAD-GAI(4)] or pAD with pBD-GoGID1 (5) did not show any LacZ activity with or without GA_3_ (Fig. 2D).

### Complementation of *Arabidopsis* mutants with *GoGID1*

**Figure 3:**
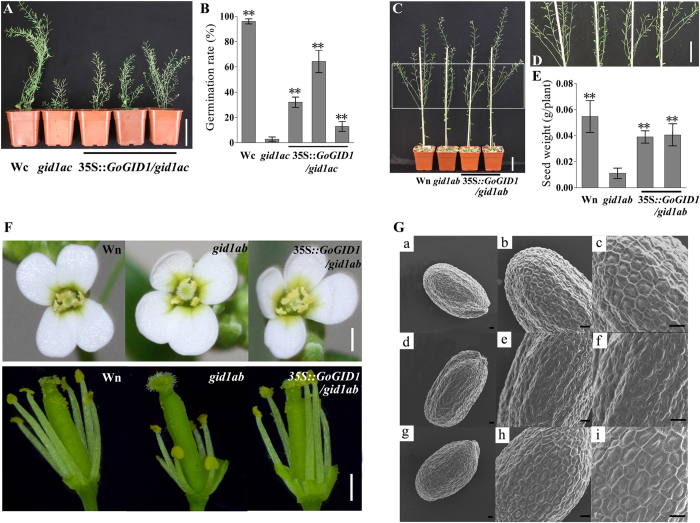
Complementation of *GoGID1* in Arabidopsis mutants. (**A**) The morphology of control, mutant *atgid1a*/*atgid1c* and transgenic *Arabidopsis*, bar: 5 cm. (**B**) The germination rate of wild type, *atgid1a*/*atgid1c* and transgenic plants. The seeds were incubated on MS agar plates at 4 °C for 2 days. WT, wild-type, Columbia background. (**C**,**D**) The morphology of control, mutant *atgid1a*/*atgid1b* and transgenic *Arabidopsis*, D is the partial detail view of C, bar: 5 cm. (**E**) The seed weight of control, *atgid1a*/*atgid1b* and transgenic *Arabidopsis*. (**F**) The flower structure of control, *atgid1a*/*atgid1b* and transgenic *Arabidopsis*. The photos were taken at full-bloom stage. Wn, Wild-type Nossen, bars: 0.5 mm. (**G**) Observation of the phenotypes and complementation of the pattern on the seed surface by scanning electron microscopy. (a–c) Control (ecotype Nossen); (d–f) *atgid1a*-*1 atgid1b*-*1* (Ns); (g–i) and *atgid1a*-*1 atgid1b*-*1* transformed with 35S:*GoGID1*. (a,d,g) The images of one seed selected at random from each pool. (b,e,h) Magnified images. (c,f,i) Further magnified images. The observations by SEM were repeated with over ten seeds of each line, and showed similar surface patterns to these figures, bars: 30 μm.

To determine whether *GoGID1* is a functional GA receptor *in vivo*, we performed a complementation assay in the *Arabidopsis* mutants. Two homozygous *Arabidopsis* double mutants, *atgid1a*/*atgid1c* and *atgid1a*/*atgid1b*, were obtained from the RIKEN Bioresource Center, and the mutant lines were confirmed by RT-PCR (Fig. S1). The mutant *atgid1a*/*atgid1c* exhibited a dwarf phenotype and low germination rate, and *atgid1a*/*atgid1b* showed a lower seed yield per plant caused by the incomplete elongation of stamens, as described by Iuchi *et al*.^13^. The *GoGID1* gene, driven by the 35S promoter, was introduced into homozygous *atgid1a*/*atgid1c*. Among the 22 kanamycin-resistant T_1_ transformants, 15 lines exhibited a partially restored phenotype, and the germination rate also increased greatly compared with that of the mutant plants (Fig. 3A,B). In addition, the 35S:*GoGID1* construct was transformed into homozygous *atgid1a*/*atgid1b* plants, and 6 of 18 transformants exhibited completely restored normal fertility (Fig. 3C-E). The length of the stamens showed no difference compared with those of the control plants (Fig. 3F). In addition, the seed surface of the mutant *atgid1a*/*atgid1b* was abnormal, with irregular swelling patterns, and this phenotypic seed surface abnormality was overcome by the overexpression of *GoGID1* (Fig. 3G). A scanning electron microscope analysis of the surface of 35S:*GoGID1*/*gid1ab* seeds showed well-ordered hexagonal patterns with volcano-shaped columellae in the center of each cell, while the surface structures of the mutant seeds were disordered (Fig. 3G). The phenotypic data and the mutant complementation analysis suggested that GoGID1 can act as a functional GA receptor in plants and has the same conserved function as AtGID.

### Expression of *GoGID1* in transgenic *Arabidopsis*

**Figure 4:**
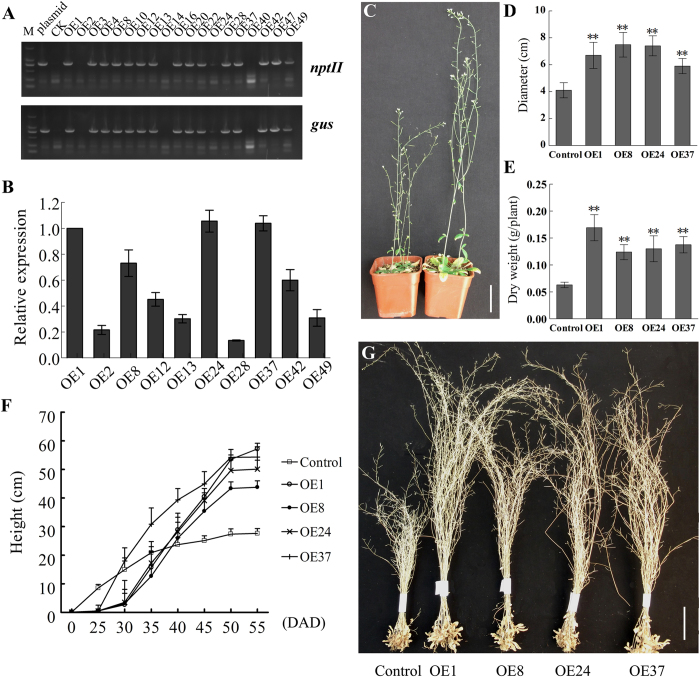
Overexpression of *GoGID1* promoted *Arabdopsis* elongation and improved biomass production. (**A**) The molecular confirmation of transgenic *Arabidopsis* lines; (**B**) The expression analysis of GoGID1 in *Arabidopsis* overexpression lines by qRT-PCR. Total RNA was extracted from 3-week-old *Arabidopsis* overexpression and WT lines (the expression of GoGID1 in WT could not be detected). The data are shown as the means ± SD of biological triplicates. (**C**) The morphology of developing control and transgenic *Arabidopsis* plants, bar: 5 cm. (**D**) Rosette diameter (means ± SD; n = 5). (**E**) The dry weight of developing control and transgenic *Arabidopsis* plants (means ± SD; n = 5). (**F**) The plant growth of transgenic lines and WT plants within 50 DAG. (**G**) The phenotype of mature control and transgenic Arabidopsis plants, bar: 5 cm.

To further test the *in vivo* function of *GoGID1*, transgenic *Arabidopsis* plants were transformed with 35S::GoGID1. Nineteen T_1_ generation lines were obtained by kanamycin-resistance selection and were confirmed to contain *GoGID1* by PCR analysis (Fig. 4A). The T_2_ generation plants were screened with kanamycin, and the segregation ratio was analyzed using a *chi*-square test. All of the homozygous T_3_ transgenic lines were tested by qRT-PCR, and the overexpression lines (OE) 1, 8, 24 and 37, having higher expression levels than the other lines, were selected for further analysis (Fig. 4B). Under normal growth conditions, the four transgenic lines had increased heights and larger rosettes when compared with the wild-type (WT) control. In addition, the flowering time of the transgenic lines was delayed by about one week, with more biomass production than WT (Fig. 4C–G). The heights of transgenic lines were increased by 59%–107% (P < 0.01) compared with WT (Fig. 4C). The transgenic lines exhibited increases of 43%–82% in rosette diameters (Fig. 4D) and of 0.96- to 1.68-fold in dry weight when compared with the control plants (Fig. 4E). Because the transgenic lines were significantly taller than the WT plants, we monitored the growth of transgenic lines and WT plants under normal conditions. At the beginning, no dramatic difference in height appeared between the transgenic lines and WT; however, after 30 days, the growth rate of the transgenic lines significantly increased compared with that of the WT. After 45 days, the WT growth plateaued, whereas the transgenic lines maintained rapid growth until 50 days (Fig. 4F).

### The effect of *GoGID1* on GA signaling in transgenic *Arabidopsis*

**Figure 5:**
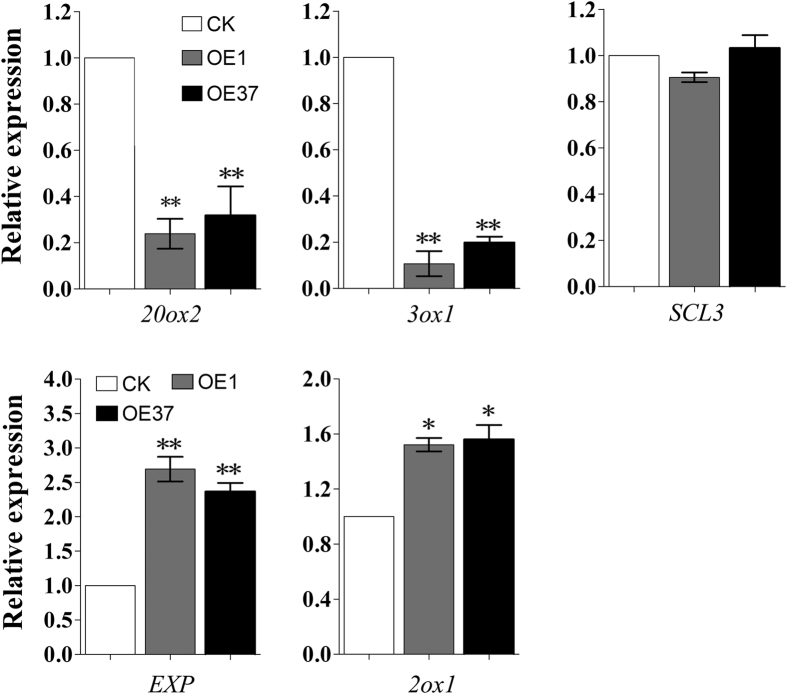
The analysis of the expression of GA metabolism and signal transduction genes involved in the GA-response in *GoGID1* overexpression plants and WT plants. RNA was isolated from 3-weeks-old plants for qRT-PCR. The data are means ± SD of biological triplicates. The expression levels were normalized to *β*-*actin*.

To better understand the function of *GoGID1* during GA signaling in plants, the expression levels of selected genes regulated by GA were analyzed in transgenic plants. The products of *AtGA20ox2* and *AtGA3ox1*, which catalyze the penultimate and final steps, respectively, in the formation of bioactive GAs, are negatively regulated by GA. The GRAS transcription regulator SCARECROW-LIKE3 (*SCL3*) is also down-regulated by GA^21^. The expansin family gene *AtEXP* and the gibberellin 2-oxidases gene *AtGA2ox1* are positively regulated by GA^22^,^23^. qRT-PCR was carried out on RNA samples isolated from transgenic and WT *Arabidopsis* leaves. As shown in Fig. 5, the mRNA expression of *AtGA20ox2* and *AtGA3ox1* was downregulated in transgenic *Arabidopsis*, while the expression of *AtEXP* and *AtGA2ox1* was upregulated compared with WT (Fig. 5). However, the mRNA level of *SCL3* did not show a significant difference between the WT and transgenic lines, potentially because there exists the functional redundancy of other SCL proteins in *Arabidopsis*^21^.

**Figure 6:**
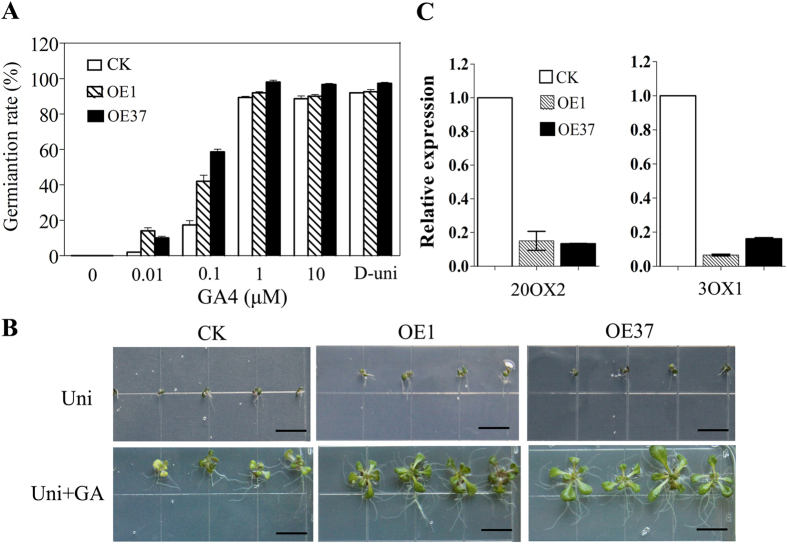
Gibberellin responses in *GoGID1* Arabidopsis transgenic lines. (**A**) Germination assay to evaluate gibberellin sensitivity. The seeds of Arabidopsis transgenic lines (OE1 and OE37) and WT control plants were germinated on 1/2 MS culture medium with or without 40 μM uniconazole or a mixture of 40 μM uniconazole and different concentrations of GA4 (0.01 to 10 μM) for 5 days. Fifty seeds were used for each treatment. The data are shown as the means ± SD of biological triplicates for each treatment. (**B**) Seedling growth response to gebberellin. The seeds of transgenic lines (OE1 and OE37) and WT were germinated in Petri dishes on 1/2 MS culture medium containing 0.1 μM uniconazole (Uni). Seven days after germination, the seedlings were treated with 10 μM GA4 for 1 h and photographed 10 days later, bar: 1 cm. (**C**) Expression level assay of GA-response genes in transgenic lines (OE1 and OE37) and WT control. RNA was isolated from 3-week-old plants treated with 10 μM GA4 for 12 h and for perform qRT-PCR analysis. The data are shown as the means ± SD of biological triplicates.

Three experiments were conducted to examine the sensitivity of the overexpression lines to GA. First, the germination rates of WT (Columbia) and transgenic seeds responding to GA_3_ were determined. Briefly, the seeds were germinated on culture medium only or on culture medium supplemented with either 40 μM uniconazole or mixtures of 40 μM uniconazole and several GA_3_ concentrations (0–10 μM) for 3 days. Seed germination was completely inhibited for all of the tested genotypes, when uniconazole only was present in the medium. When 0.01 μM GA_3_ was added, the germination rates of WT and transgenic lines were recovered to a different extent, with the transgenic lines having higher germination rates than the WT seeds. The transgenic lines remained more sensitive than the WT when 0.1 μM GA_3_ was added to the medium, whereas with GA supplements equal to or above 1 μM, the germination rates of the transgenic seeds, independent of line, were almost equivalent to that of the WT (Fig. 6A).

Second, the growth responses of transgenic and WT seedlings to GA were determined. The seeds of OE1, OE37 and WT were germinated in Petri dishes on 1/2 Murashige and Skoog (MS) medium containing uniconazole. The seedlings were treated with GA_3_ 7 days after germinating. A reduced dwarfing effect of uniconazole was observed in the transgenic lines compared to the WT. The addition of 0.1 μM GA partially rescued the seedling growth. The recovery effect on OE1 and OE37 growth was stronger than on WT growth (Fig. 6B).

Finally, the expression levels of genes that are regulated by GA were analyzed in the transgenic plants. *GA20ox2* and *GA3ox1* were selected for this experiment. *GA20ox2* and *GA3ox1* had lower expression levels in the transgenic lines than in the WT plants following the application of 10 μM GA_3_ (Fig. 6C).

Based on these results, we concluded that the overexpression of the GoGID1 increased the sensitivity of *Arabidopsis* to GA.

### Ectopic expression of *GoGID1* increased the biomass in transgenic alfalfa

**Figure 7:**
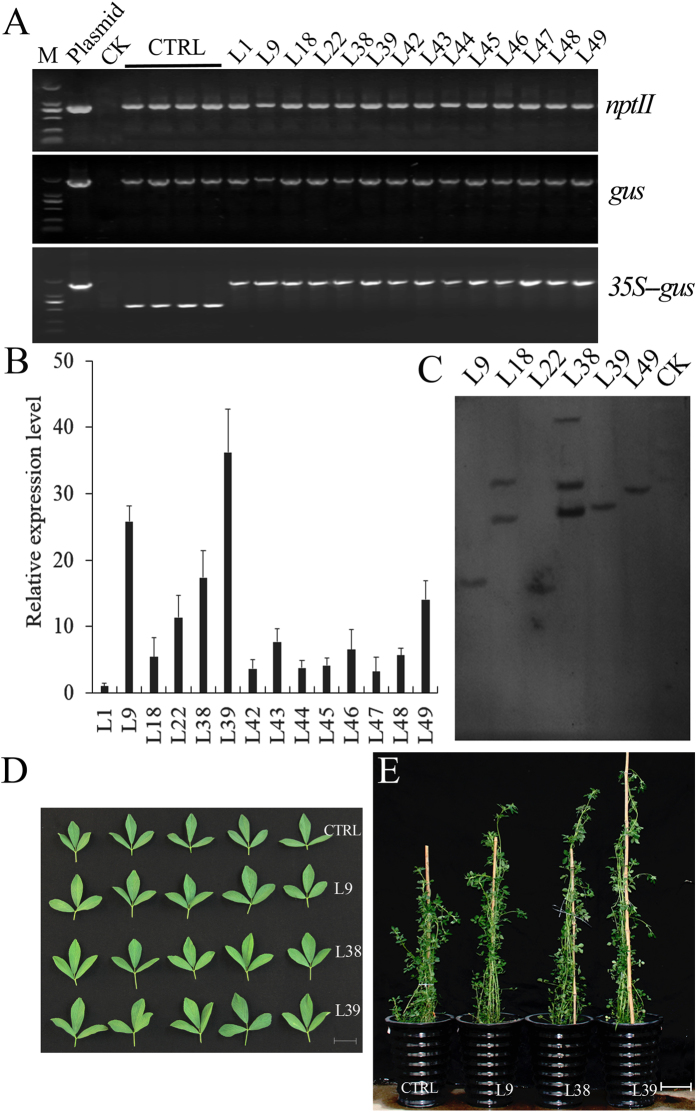
Phenotypic appearance of transgenic alfalfa and WT control plants under normal growth conditions. (**A**) The PCR analysis of regenerated alfalfa plants, the positive control (pBI121-*GoGID1* plasmid), the negative control (wild-type), and empty vector control (CTRL). The sizes of the DNA fragments were 795 bp for *NPT*II (upper), 1812 bp for *GUS* (middle), 553 bp for part of the 35S-GUS sequence, and 1585 bp for the *GoGID1* gene plus 553 bp. (**B**) The transcript abundance of *GoGID1* in transgenic plants was measured by quantitative RT-PCR. The alfalfa actin gene was used as a reference for normalization. (**C**) Southern-blot analysis of *Hin*dIII-digested genomic DNA from leaves of the wild-type and *GoGID1*-over-expressing lines. The DNA was probed with the 795-bp *NPT*II fragment from the pBI121 vector. (**D**) The leaf phenotypes of the control and transgenic lines, bar: 2 cm; (**E**) The gross morphology of the transgenic lines and WT control, bar: 10 cm.

The constructed vector 35S::GoGID1 was introduced into alfalfa by *Agrobacterium*-mediated transformation. Plantlets with well-developed roots were transplanted into soil. The greenhouse-grown alfalfa plants were subjected to PCR screening using different primers, and distinct bands of expected sizes were obtained from 50 transgenic events (Fig. 7A).

A Southern-blot hybridization analysis confirmed that the transgene was stably integrated in the alfalfa genome (Fig. 7C). A qRT-PCR analysis revealed that the transgenic lines L9, L38 and L39 had higher expression levels than the other lines, and these three lines were selected for further analysis (Fig. 7B). To evaluate the effects of *GoGID1* expression on alfalfa development and biomass, the following traits were measured: plant height, dry matter biomass, leaf length, leaf width, branch number, internode number and flowering time. Five-month-old alfalfa plants were used to measure plant height, internode number, and leaf length and width. Six plants per line were measured as replicates. No significant differences were observed between the empty vector control and WT plants with regards to these seven parameters. The selected transgenic lines exhibited similar phenotypic traits, with increased heights, greater biomass and larger leaves when compared with WT plants (Table 1). The transgenic lines showed a 0.20- to 0.35-fold increase in plant height compared with controls (P < 0.01). The dry weights of the transgenic plants were 14.9–46.3% higher than those of the control plants. The leaf lengths, leaf widths and numbers of branches in the transgenic lines also significantly increased, compared with those of the control plants, and they had larger leaves and flowered later (Table 1). However, the positive effect on branch numbers that occurred in the initial establishment of young plantlets in the growth chamber did not result in a significant affect at later stages (data not shown). The effect on branch number varied with the stage of plant development. No differences were observed in internode numbers between the transgenic lines and the control plants.

### Nutritional qualities of transgenic alfalfa were not compromised

The forage quality of the transgenic alfalfa plants was also evaluated by measuring crude protein (CP), neutral detergent fiber (NDF), acid detergent fiber (ADF) and acid detergent lignin. No differences in CP and NDF values were found between the transgenic lines and control plants. Among the three transgenic lines tested, L39 showed a slight increase in ADF (Table 2). Thus, GID overexpression could sharply increase the biomass of alfalfa, suggesting that a molecular modification using the GA receptor gene is a feasible approach for alfalfa biomass improvement.

## Discussion

Plant biomass yield is determined by a number of factors that regulate plant developmental processes, such as vegetative meristem activities, cell elongation, photosynthetic efficiency and secondary wall biosynthesis^24^. The potential for improving plant biomass production has not yet been extensively explored since the traditional breeding of crop plants (maize, rice, wheat and soybean) has focused on the selection of high grain-yield traits^24^. The aerial part of alfalfa is most useful for livestock feed; therefore, the biomass yield is always the most important trait in breeding and is an economics-driven target. The genetic improvement of alfalfa, especially of quantitative traits like biomass, has been slow due to certain plant characteristics, including the necessity of flower tripping by insects for pollination, self-incompatibility, inbreeding depression and heterozygosity^25^. GAs play important roles in plant growth and development, including the regulation of cell elongation, which is crucial for biomass yield. The regulation of GA processes is critical to agriculture, including ‘Green Revolution’ genes, making the GA pathway a prime target for improving yield^26^. Although a number of reports have indicated the importance of GIDs in the regulation of plant development^4^,^8^,^13^,^18^, little is known about their functions in biomass production and potential practical applications, especially in forage crops. In this study, a GID-like gene isolated from *G. orientalis* was overexpressed in alfalfa. The biomass of transgenic alfalfa increased 14.9–46.3%, without sacrificing quality, compared with that of WT. To our knowledge, this is the first time that a GA-signaling gene was identified and applied in the biomass improvement of alfalfa.

GID proteins are important factors in GA perception in both dicotyledonous and monocotyledonous plants^27^. The GA-dependent interaction between GIDs and DELLA is an important step in GA-signal transduction^28^. In this study, yeast cells carrying the plasmid pBD-GoGID1 with either pAD-RGA or pAD-GAI grew well on QDO + X-a-Gal agar plates in the auxotrophic screen and had LacZ activity in the presence of GA_3_, while the yeast cells containing the plasmid combinations of pBD with DELLA did not show any LacZ activity with or without GA_3_ (Fig. 3). Thus, *GoGID1* interacts with AtRGA and AtGAI in a GA-dependent manner. Furthermore, the overexpression of *GoGID1* in *Arabidopsis* double mutants (*atgid1a*/*atgid1c* and *atgid1a*/*atgid1b*) partially or completely rescued the dwarf, low germination, sterile and irregular seed surface phenotypes. Therefore, GoGID1 is a biologically functional GA receptor.

Additionally, the overexpression of *GoGID1* led to increased plant heights and improved biomass production in transgenic *Arabidopsis* plants. The overexpression of GID in rice^4^, aspen^18^ and tobacco^8^ also indirectly/directly supports this result. In the present study, the seed weights of transgenic lines were increased compared with those of the control, which is unlike the observations noted rice^4^ and tobacco^8^. This result suggested that GID proteins from different organisms might regulate specific phenotypic consequences with regards to seed weight.

Because *GoGID1* is a functional GA receptor, we examined the sensitivity to GA of transgenic and WT *Arabidopsis* plants. The supplementation of growth medium with 40 μM uniconazole totally inhibited seed germination in transgenic and WT *Arabidopsis*. However, after adding GA, germination was rescued and was significantly higher in transgenic plants than in WT plants. In addition, the expression levels of *GA20ox2* and *GA3ox1*, which are negatively regulated by GA, were significantly decreased in transgenic lines compared with WT. Thus, our data showed that the overexpression of *GoGID1* increased the sensitivity of *Arabidopsis* to GA.

Spurred by the success in *Arabidopsis*, the potential application of *GoGID1* overexpression was explored in alfalfa. As expected, a similar phenotype was observed when *GoGID1* was expressed in alfalfa, with the transgenic plants exhibiting 0.2- to 0.35-fold increases in plant heights. Plant height is the most important of the biomass yield components^15^. Furthermore, the overexpression of *GoGID1* in alfalfa promoted taller stems, larger leaves and later flowering, which contributed to the 14.9%–46.3% increase in biomass. Overall, *GoGID1* regulated the biomass of alfalfa by regulating relevant factors, including plant height, stem length, leaf width and flower time.

GAs have long been reported to be involved in flowering and flower development. Manipulating GA signalling affects not only plant growth and morphology but also flowering time. A GA overproduction phenotype is associated with early flower induction^17^. The overexpression of different GA20-oxidases in transgenic *Arabidopsis* plants resulted in early flowering^29^. In the present study, the flowering times of both transgenic *Arabidopsis* and alfalfa plants were delayed. When WT *Arabidopsis* plants had already begun blossoming, the transgenic lines were still in a vigorous growth period. The flowering time of transgenic alfalfa was delayed by 3–4 days, and the overexpression lines had longer vegetative stages. GA20-oxidases catalyze the penultimate step in bioactive GA synthesis, while GID is the GA-receptor that receives the GA signal and launches the GA response. This indicated that GA biosynthesis-related genes and regulatory genes related to GA signaling have opposite influences on flowering time.

The expression of 35S-GoGID1 in wild-type *Arabidopsis* not only delays flowering but also produces a larger rosette. This was contradictory to the results obtained by Willige *et al*.^9^, who reported that 35S:GID1-GFP *Arabidopsis* plants flowered earlier than WT plants and consequently had a smaller rosette. This discrepancy may be attributable to the uses of different GID1 homologs in these studies. As mentioned in the introduction, *Arabidopsis* carries three GA receptors (GID1a, GID1b, GID1c), and it was found that the three *GID1* genes have redundant functions in mediating GA responses. *AtGID1a* and *AtGID1b* are involved in flower development, while *AtGID1a* and *AtGID1c* are vital for stem elongation. However, in our experiments, the *35S:GoGID1* could only partly rescue the *Arabidopsis* mutant. The result indicates that *GoGID1* still has some functions that are different from the *Arabidopsis* homologs. Therefore, the overexpression of GoGID1 in the WT *Arabidopsis* background may lead to a competition with endogenous GID1 genes in the regulation of the flowering time.

High biomass and quality are two important target traits in forage production. The overexpression of *GoGID1* significantly increased the biomass of transgenic alfalfa; therefore, we determined whether the transformation affected quality of the alfalfa. A traditional forage quality analysis determines CP, ADF, NDF, ash and other extract levels. In the present study, the overexpression of *GoGID1* did not negatively impact the forage quality traits of CP and NDF. The transgenic OE39 line exhibited a slight increase in ADF level compared with WT plants, whereas the two other lines tested showed no significant differences in ADF. By selection, transgenic lines with a balance between biomass and quality can be obtained. Thus, it is possible to improve the biomass of alfalfa using transgenic technology and to establish GID as a candidate gene, which could be used not only in alfalfa but also potentially in other important forage crops.

In conclusion, *GoGID1* encodes a biologically functional GA receptor, which can interact with the GA-related RGA and GAI proteins. *GoGID1* was introduced into the *Arabidopsis* gid1-1 mutant background and functioned as a GA receptor in planta based on growth restoration and other phenotypes. The ectopic expression of *GoGID1* in *Arabidopsis* and alfalfa increased plant heights and improved biomass yields. Thus, it is possible to genetically engineer alfalfa having a higher growth rate and improved biomass simply by overexpressing a single gene, which would be more efficient than conventional breeding programs. Similar strategies could potentially be applied to improve the yield of other forage crops.

## Methods

### Yeast two-hybrid assay

The Yeast Two-hybrid System (Clontech, New Jersey, USA) was used in the present study. The entire coding region of *GoGID1* was ligated into the pGBT9-DNA-BD shuttle vector using the *Eco*R I and *Pst*I sites to generate the bait plasmid. Similarly, the entire coding region of *AtGAI* and *AtRGA* were independently ligated into the pGADT7-AD vector using the *Nde*I and *Bam*H I sites to generate the prey plasmid.

Vector cassettes for GADT7-T/pGBKT7-53 and pGADT7-T/pGBKT7-Lam were used as positive and negative controls, respectively. The *Saccharomyces cerevisiae* strain Y2H Gold or Y187 was used. The plate media were supplemented with 10^–4 ^M GA_3_ to determine GA dependency. The experiments were conducted three independent times, producing similar results each time.

### Plant materials and growth conditions

*A. thaliana gid* mutants were described previously^13^, and the double-knockout mutants *atgid1a*-*2*/*atgid1c*-*1* (Resource number Psi00016) and *atgid1a*-*1*/*atgid1b*-*1* (Resource number Psi00017) were obtained from the RIKEN Bioresource Center (Japan). The WT *Arabidopsis* and overexpression lines were sterilized and transferred to 1/2 MS agar plates at 4 °C for 2 days, and incubated at 22 °C under constant fluorescent light for 10 to 14 days. The *Arabidopsis* seedlings were transferred to soil and grown at 22 °C under fluorescent light conditions (16-h/day).

A widely used and highly productive alfalfa (*Medicago sativa* cv. Zhongmu No. 1), was used for genetic transformation and biomass improvement. All of the transgenic plants were propagated from the cuttings of young shoots. Six copies of each line were grown in 2-gallon pots and randomly distributed into six blocks.

Seeds from the *Arabidopsis GoGID1* overexpression and WT lines were sterilized, vernalized and germinated on plates containing sterile 1/2 MS medium, 0.8% (w/v) agar and 1.5% (w/v) sucrose. To determine the seed germination responses to GA, the seeds were germinated on either 1/2 MS medium, a medium supplemented with 40 μM uniconazole or media containing mixtures of 40 μM uniconazole and different concentrations of GA3 (0–10 μM) for 3 days. The germinated seeds were counted to calculate the germination rate. Each treatment was performed with biological triplicates, each containing 50 seeds. To examine seedling growth responses to GA, 1/2 MS medium with 0.1 μM uniconazole was used when germinating the transgenic and WT seeds. The seeds were treated with 10 μM GA_3_ for 1 h, 7 days after germination. The seedlings were grown in a growth room under a controlled temperature (22 °C) and long (16-h light/8-h dark) photoperiods. The phenotypes of the seedlings were photographed after 10 days. Each treatment was performed in triplicate.

### Gene constructs and plant transformation

The coding sequence of *GoGID1* was amplified by PCR using the following primers: *GoGID1*121-F, 5′-GCTCTAGAATGACTGGAAGTAATGAAGTCAACC-3′ (*Xba*I site underlined), *GoGID1*121-R, 5′-CGGGATCCACAGTTAGGGTGCACAAAG-3′ (*Bam*HI site underlined). The PCR product was digested by *Xba*I/*Bam*HI and ligated into the pBI121 vector. The construct was confirmed by sequencing and transformed into *Agrobacterium* strain GV3101 by the freeze-thaw method of plant transformation^30^. The *Arabidopsis* transformation was conducted by floral dipping^31^.

Transgenic plants containing a single-copy of the *GoGID1* gene were identified by Southern hybridization analysis. The T_3_ generation of homozygous *Arabidopsis* lines was obtained by kanamycin selection and PCR screening. High embryogenic calli of the alfalfa cultivars were used for *Agrobacterium*-mediated transformation following a previously described procedure^32^. Non-transformed alfalfa plants that were taken through somatic embryogenesis in parallel with the transgenic plants were used as controls.

### PCR and Southern blot analysis of transgenic plants

Genomic DNA was isolated from transgenic and non-transgenic plants using the CTAB method^33^. The transgenic alfalfa plants were identified by PCR with specific *npt*II, gus or 35S-gus primers (Table S1). The expected PCR products were 795 bp, 1,812 bp and 553 (or 1,585) bp, respectively. For the Southern hybridization analysis, DNA was digested with the restriction enzyme *Hin*dIII, which cuts once at a site outside of the *npt*II gene sequence in the binary vector. Then, 40 μg of DNA from each sample was digested, electrophoresed on 0.8% agarose gels and transferred to positively charged nylon membranes by alkaline capillary blotting. The hybridization probe (*npt*II) was labelled with digoxigenin (DIG) by PCR, and the hybridization was carried out using the DIG Luminescent Detection Kit (Roche).

### qRT-PCR

Total RNA was isolated by TRIzol extraction (Invitrogen, California, USA), and all of the samples were treated with DNase to remove DNA contamination. Then, 1 μg of the total RNA was reverse transcribed into cDNA using SuperScript III (Invitrogen, USA). The cDNA (5 ng) was used in a 20 μl reaction in an ABI 7500 Real Time PCR System (Bio-Rad, California, USA) using SYBR Green PCR Master Mix (Takara, Japan). The gene-specific primers used are listed in Table S1. The relative gene expression levels were calculated by the 2^−ΔΔCt^ method^34^. The reference genes *GoActin, AtActin* and *MsActin* were used to normalize the gene expression levels in *G. orientalis, Arabidopsis* and alfalfa, respectively. The experiments were performed using three biological replicates.

### Measurement of plant height and dry matter contents

The data on plant heights and numbers of branches per meter were recorded by selecting 10 plants randomly from each plot. The plant height was measured using a measuring tape from the ground level to the highest leaf tip. For the dry matter content, plant samples were dried in the shade and then transferred to an electric oven at 70 °C until a constant weight was reached.

### Determination of forage quality

Vegetatively propagated alfalfa cuttings were grown in parallel in 1-gallon pots in the greenhouse. Aerial portions were harvested at the early bud stage to ensure the materials were developmentally matched and dried in a 50 °C oven for at least 72 h. The samples were then ground in a Thomas-Wiley model 4 Laboratory Mill (Lehman Scientific, Wrightsville, PA, USA) with 1-mm sieves. ADF and NDF were estimated using standard protocols.

To determine the *in vitro* digestibility, the ground samples were dried at 105 °C for 6 h before calculating their pre-extraction dry weights. The same procedure was used to obtain post-extraction dry weights. A digestibility analysis (0.5 g samples) was performed using F57 filter bags and a DAISY incubator (ANKOM Technology) following the manufacturer’s instructions. Digestibility was calculated based on the dry weight differences of samples before and after digestion^35^.

## Additional Information

**How to cite this article**: Wang, X. *et al*. Functional characterization of a gibberellin receptor and its application in alfalfa biomass improvement. *Sci. Rep.* **7**, 41296; doi: 10.1038/srep41296 (2017).

**Publisher's note:** Springer Nature remains neutral with regard to jurisdictional claims in published maps and institutional affiliations.

## Supplementary Material

Supplementary Data

## Figures and Tables

**Table 1:** Morphological characterization of control and transgenic alfalfa plants.

Lines	Plant height (cm)	Dry weight (g)	Leaf length (mm)	Leaf width (mm)	Number of branches	Rang of internode number	Flowering time (day)
Control	70.75 ± 5.74	6.37 ± 1.22	22.71 ± 1.63	9.74 ± 1.19	7.25 ± 0.96	14–16	25 ± 2
L9	84.50 ± 2.52**	7.99 ± 0.34**	25.54 ± 1.73**	11.89 ± 0.87**	9.75 ± 0.96**	14–16	29 ± 2
L38	95.50 ± 4.93**	9.32 ± 0.78**	23.50 ± 1.70**	10.50 ± 0.78**	9.75 ± 1.71**	13–15	28 ± 2
L39	87.00 ± 6.68**	8.30 ± 0.96**	24.67 ± 1.23**	11.22 ± 0.85**	9.25 ± 1.26**	13–17	29 ± 2

Values are mean ± SD (n = 6). One or two asterisks indicate significances corresponding to P < 0.05 or 0.01 (one way ANOVA, Dunnett’s test), respectively.

**Table 2:** Forage quality analysis of transgenic alfalfa plants.

	CP (%/DW)	NDF (%/DW)	ADF (%/DW)
Control	16.67 ± 1.13	42.72 ± 0.97	31.72 ± 0.66
L9	16.06 ± 0.39	42.13 ± 0.68	30.87 ± 0.54
L38	16.93 ± 0.87	42.53 ± 0.61	31.38 ± 0.46
L39	16.75 ± 0.79	44.32 ± 1.90	33.30 ± 1.08

CP, crude protein; ADF, acid detergent fiber; NDF, neutral detergent fiber; values are mean ± SD (n = 3). One or two asterisks indicate significances corresponding to P < 0.05 or 0.01 (one way ANOVA, Dunnett’s test), respectively.
