# Supplementary material for: Functional characterization of a gibberellin receptor and its application in alfalfa biomass improvement
Source: Sci Rep. 2017 Jan 27;7:41296. doi: 10.1038/srep41296 (PMC5269710; doi:10.1038/srep41296)
Supplement: Supplementary Data [file srep41296-s1.pdf]

Title： Functional characterization of a gibberellin receptor and its application in alfalfa biomass improvement

Xuemin Wang[1#], Jun Li[2#], Liping Ban[3], Xinming Wu[4], Yunqi Wang[4], Yudi Wu[1], Hongyu Wen[1], Vladimir Chapurin[5], Nikolay Dzyubenko[5], Zhiyong Li[2], Zan Wang[1*], Hongwen Gao[1*]

1. Institute of Animal Science, Chinese Academy of Agricultural Sciences, Beijing 100193, China.
2.Institute of Grassland Research, Chinese Academy of Agricultural Sciences, Hohhot 010010, China
3. College of Animal Science and Technology, China Agricultural University, Beijing 100193, China.
4.Animal Husbandry and Veterinary institute, Shanxi Academy of Agricultural Sciences, Taiyuan 030032, China.
5. N.I.Vavilov All-Russian Research Institute of Plant Industry, St. Petersburg 190000, Russia

[#] These authors contributed equally to this work

* Corresponding author E-mail: gaohongwen@263.net; wangzan@caas.cn.

Supplementary Figure S1

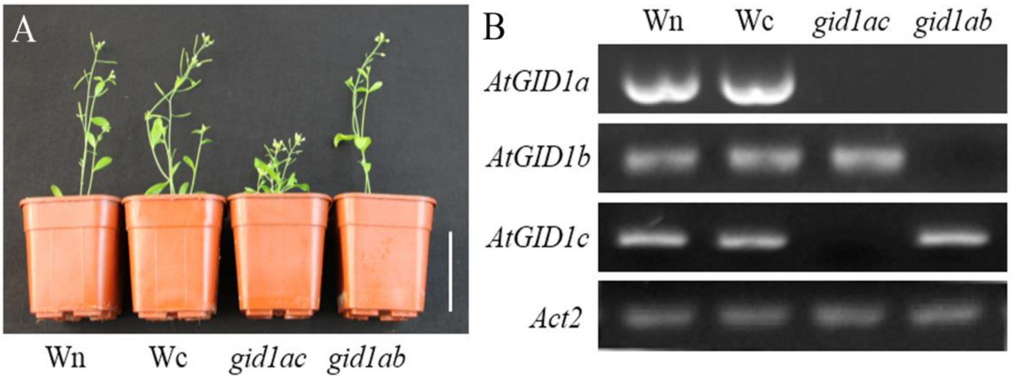

Figure S1 Identification of mutants in phenotype and expression level.
(A)Phenotype of wild-type and mutants; bar: 5 cm.
(B)Identification of homozygotes of the mutants lines by RT-PCR. ACTIN2 gene was used as control.

Table S1. Primers list

Primers	Sequence (5′→3′)	Application
GID-PBI-F	GCTCTAGAATGACTGGAAGTAATGAAGTCAACC	Vector construction
GID-PBI-R	CGGGATCCACAGTTAGGGTGCACAAAG	Vector construction
NPT-PBI-F	ATGATTGAACAAGATGGATTGCACGCAG	*Genotyping*
NPT-PBI-R	TCAGAAGAACTCGTCAAGAAGGCGA	*Genotyping*
GUS-PBI-F	ATGTTACGTCCTGTAGAAACCCCAACC	*Genotyping*
GUS-PBI-R	TCATTGTTTGCCTCCCTGCTGC	*Genotyping*
35S-PBI-F	GGTGGCTCCTACAAATGCCA	*Genotyping*
GUS-PBI-R2	GAAACGCAGCACGATACGC	*Genotyping*
AtGA2ox1-F	TGAGGACGAGAGGTTGTACGA	*Q-PCR*
AtGA2ox1-R	TCCTTTCGAATTGTTGAAGCC	*Q-PCR*
AtGA20ox2-F	ACCGAGACTATTTCCGAGGATT	*Q-PCR*
ATGA20ox2-R	TGTTTGGCATGGAGGATAATG	*Q-PCR*
AtEXP-F	ACCTTCTTGTTTATTGCTACCCTTG	*Q-PCR*
AtEXP-R	AAGCATCACCACCACCGTAGAATG	*Q-PCR*
At3OX1-F	ATTCCTTTGGGGTCCGCAATCT	*Q-PCR*
At3OX1-R	GGTATAGAGGCGATTCAACGGGACT	*Q-PCR*
AtActin-F	CGCCATCCAAGCTGTTCTC	*Q-PCR*
AtActin-R	TCACGTCCAGCAAGGTCAAG	*Q-PCR*
MsActin-F	GAGCGTTTCCGTTGTCCTGA	*Q-PCR*
MsActin-R	AGGTGCTGAGGGAAGCCAAA	*Q-PCR*
GoActin-F	GGACAAGTTATC ACAATCGG	*Q-PCR*
GoActin-R	TCAGCAATACCTGGAAACATAG	*Q-PCR*
GoGID-F	GCAAAGTCCCAGCCAATACAA	*Q-PCR*
GoGID-R	CGGCAGAAAGTGTCGTAAATAGC	*Q-PCR*
